# Gene expression differences between PAXgene and Tempus blood RNA tubes are highly reproducible between independent samples and biobanks

**DOI:** 10.1186/s13104-017-2455-6

**Published:** 2017-03-23

**Authors:** Anne Heidi Skogholt, Einar Ryeng, Sten Even Erlandsen, Frank Skorpen, Svanhild A. Schønberg, Pål Sætrom

**Affiliations:** 1 Department of Laboratory Medicine, Children’s and Women’s Health, Faculty of Medicine, Norwegian University of Science and Technology-NTNU, 7491 Trondheim, Norway; 2 Department of Cancer Research and Molecular Medicine, Faculty of Medicine, Norwegian University of Science and Technology-NTNU, 7491 Trondheim, Norway; 3 Department of Computer and Information Science, Faculty of Information Technology, Mathematics and Electrical Engineering, Norwegian University of Science and Technology-NTNU, 7491 Trondheim, Norway; 4 Bioinformatics Core Facility-BioCore, Faculty of Medicine, Norwegian University of Science and Technology-NTNU, 7491 Trondheim, Norway

**Keywords:** miRNA, RNA-isolation, Blood samples, Sampling systems, Microarray

## Abstract

**Background:**

Gene expression profiling from blood is sensitive to technology choices. For example, the main blood RNA collection systems—the PAXgene and Tempus tubes—differently influence RNA expression signatures. The aim of this study was to establish a common RNA isolation protocol for these two systems and investigate if it could reduce the differences in gene expression between them.

**Results:**

We collected identical blood samples on the PAXgene and Tempus systems and retrieved blood samples from two independent biobanks—NOWAC and HUNT3—which are based on PAXgene and Tempus, respectively. High-quality RNA was extracted from both sampling systems by using their original protocols and our common modified protocol, and were profiled on Illumina microarrays. Regardless of the protocol used, we found most of the measured transcripts to be differently affected by the two sampling systems. However, our modified protocol reduced the number of transcripts that were significantly differentially expressed between PAXgene and Tempus by approximately 50%. Expression differences between PAXgene and Tempus were highly reproducible both between protocols and between different independent sample sets (Pearson correlation 0.563–0.854 across 47323 probes). Moreover, the modified protocol increased the microRNA output of the system with lowest microRNA yield, the PAXgene system.

**Conclusions:**

Most transcripts are affected by the choice of sampling system, but these effects are highly reproducible between independent samples. We propose that by running a control experiment with samples on both systems in parallel with biologically relevant samples, researchers may adjust for technical differences between the sampling systems.

**Electronic supplementary material:**

The online version of this article (doi:10.1186/s13104-017-2455-6) contains supplementary material, which is available to authorized users.

## Background

Blood-based gene expression profiling is a valuable utility in biomarker analysis. Samples of blood are easily available, essentially non-invasive, and can be collected at a low cost, all of which makes blood samples attractive for diagnostic purposes. Peripheral blood is the main route for transportation of immune cells and thereby provides a window for monitoring activity of the immune system [1–4]. Indeed, presence of disease [5–10], prognostic information [11, 12] and effect on therapeutic response [13] have been found to be reflected in the gene expression pattern of blood cells.

The two main commercial systems for the isolation of high-quality RNA from blood are the PAXgene Blood RNA System (PreAnalytiX QIAGEN/BD, Hombrechtikon, Switzerland) and the Tempus Blood RNA System (Applied Biosystems, Foster City, CA, USA). These two systems use proprietary reagents that intend to stabilize RNA and ensure gene expression profiles that reflect the blood’s state at the moment of sampling. Even though both sampling systems have the same purpose, they result in gene expression profiles that differ between the systems [14–16]. The recommendation from earlier studies is to avoid the mixed use of these sampling systems in the same experiment [14–16]. Unfortunately, this strategy restricts studies that may be based on combining existing biobanks to biobanks that use identical sampling systems. Moreover, when assessing reproducibility of blood gene expression profiles, one should ideally include and test independent cohorts irrespective of their sampling systems.

As the PAXgene and Tempus systems recommend different protocols to isolate RNA (PreAnalytiX, and Applied Biosystems, respectively), we set out to investigate if the use of a common protocol could reduce some of the differences between the two sampling systems. We developed a protocol that could isolate both microRNA (miRNA) and messenger RNA (mRNA) into the same batch, and that could be used with both PAXgene and Tempus tubes. Although this protocol still results in differences in gene expression profiles between the two sampling systems, we show that the differences are reduced compared to the original protocols and that the differences are reproducible between cohorts. These results suggest that our setup with a control experiment can be used to estimate and correct for the effects of the technical differences between the two sampling systems.

## Results and discussion

Previous studies have shown that the two RNA isolation systems result in gene expression profiles that may differ significantly [14–16]. As the differences may relate to differences in the composition of the RNA-stabilizing solutions or in the isolation protocols, we wanted to test whether a common protocol could extract both mRNA and miRNA, and at the same time reduce the technical differences. We developed a protocol that combines elements from the original PAXgene and Tempus protocols, and also includes the final steps from the mirVana RNA isolation protocol (Life Technologies, Carlsbad, CA, USA, Part Number AM1560) for isolating both mRNA and miRNA.

Briefly, the modified protocol processed the stabilized blood by removing the stabilization buffer in the tubes by the aid of the Tempus Spin RNA Isolation Kit protocol until a pellet was produced. The pellet was further purified by the use of the PAXgene protocol. The RNA was then rinsed by the use of the mirVana miRNA Isolation Kit (Life Technologies), and finally eluted in RNase free water (Tempus kit, Applied Biosystems). We considered but eventually decided not to include globin RNA reduction, as this process would have introduced more steps in the protocol which in turn may lead to more variation in the results [14, 15].

**Fig. 1:**
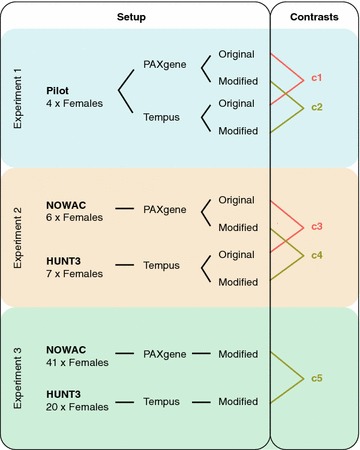
Study design. Differences in gene expression between PAXgene and Tempus were investigated in three experiments. In experiment 1 (*light blue*), four volunteers donated blood samples on PAXgene and Tempus tubes and RNA was isolated with both the original protocols and the modified protocol. Paired statistical analyses identified differences between PAXgene and Tempus for the original protocols (contrast 1) and for the modified protocol (contrast 2). In experiment 2 (*light orange*), RNA was isolated with both the original protocols and the modified protocol from two different biobanks—NOWAC and HUNT3—which had samples on PAXgene and Tempus tubes, respectively. Non-paired statistical analyses identified differences between PAXgene and Tempus for the original protocols (contrast 3) and for the modified protocol (contrast 4). In experiment 3 (*light green*), RNA was isolated with the modified protocol from a larger set of samples from NOWAC and HUNT3 and a non-paired analysis was performed (contrast 5). Comparisons between PAXgene and Tempus based on the original protocols are highlighted in orange (contrasts 1 and 3), whereas comparisons based on the modified protocol are highlighted in *olive green* (contrasts 2, 4, and 5)

The following sections describe the comparisons of the original and modified protocols in terms of RNA yield, quality, and gene expression profiles. We used an experimental design consisting of three experiments that allowed us to assess both the differences in gene expression produced by the protocols, as well as the reproducibility of these differences between different experiments and cohorts (Fig. 1; Table 1).

**Table 1:** Contrasts produced from comparing expression profiles in PAXgene and Tempus tubes

Contrast	Experiment	Protocol	Sign. probes*	Sign. Transcripts*
c1	1	Original	3143	2883
c2	1	Modified	1540	1469
c3	2	Original	2236	2068
c4	2	Modified	1440	1346
c5	3	Modified	7142	6250

“Contrast” enumerates the five statistical comparisons specified by “Experiment” and “Protocol”; see Fig. 1. “Sign. probes” are the number of probes on the Illumina microarray (total probes: 47323) with significantly different signals in PAXgene compared with Tempus (* p < 0.05); “Sign. Transcripts” are the number of distinct transcripts targeted by the significant probes

### RNA quality and quantity

#### RNA yield and RIN values

**Fig. 2:**
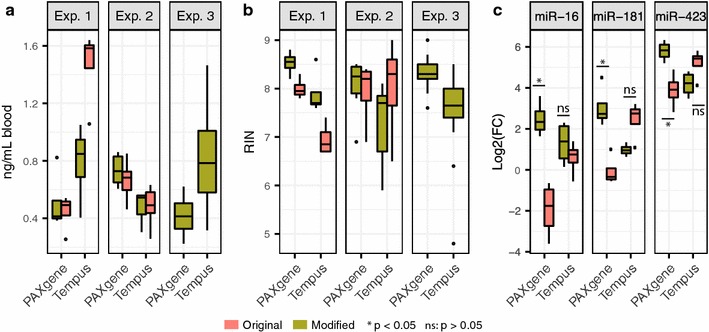
RNA yield and quality. Concentration (**a**), quality (RIN) (**b**), and miRNA levels (**c**) for RNA isolated from Tempus and PAXgene tubes with the original and modified protocols. Concentration and quality measurements are from all three experiments; miRNA levels are miR-16, miR-181, and miR-423 expression levels from experiment 1. The graphs are *box-plots* of the data, where the *box* with *horizontal black line* shows the first and third quartiles and the median; the *whiskers* show 1.5 times the interquartile range; and the points show outliers

High quality RNA was obtained with all protocols from both PAXgene and Tempus tubes (Fig. 2; Additional file 1). The overall total RNA concentration obtained from the PAXgene and Tempus tubes when processed with the original protocols varied between the sampling systems (0.55 ± 0.002 ng/mL blood and 0.98 ± 0.1 ng/mL blood, respectively), though the RNA concentration obtained from the Tempus tubes varied extensively between the experimental cohorts (compare Exp. 1 and Exp. 2, Fig. 2a). With the modified protocol, the RNA concentration obtained from PAXgene tubes was essentially similar to that of the original protocol (0.55 ± 0.06 ng/mL blood), whereas the concentration from Tempus tubes was somewhat reduced compared to the original protocol (0.69 ± 0.11 ng/mL blood, all 3 experiments, Fig. 2a). The RNA quality of the samples was generally high (RIN = 7.98 ± 0.27), with some variation (6.96–8.53) between protocols and tubes (all 3 experiments, Fig. 2b).

#### miRNA

To detect and quantify miRNA expression, we ran TaqMan qPCR against hsa-miR-16-5p, hsa-miR-181-5p, and hsa-miR-423-3p on all samples from experiment 1 (Fig. 2c). With the original protocols, compared with the Tempus samples, the PAXgene samples had significantly lower levels of miR-16 (log_2_ fold change (logFC) = −2.52, p = 0.01) and miR-181 (logFC = −2.51, p = 0.008), and reduced levels of miR-423 (logFC = −1.31, p = 0.07). Using the modified protocol, the level of miRNAs isolated from the PAXgene tubes increased significantly for all three miRNAs compared with the original protocol (Fig. 2c). For the Tempus tubes, the level of miRNAs isolated with the modified protocol compared with the original protocol differed depending on the miRNA assayed, though none of the differences were significant (Fig. 2c).

### Gene expression

#### Principal components analysis

To explore the main sources of variation in the gene expression profiles from the samples, we used principal components analysis (PCA, Additional file 2A). The main difference observed was between the first experiment and the two other experiments (PC1, Additional file 2A). This difference is likely a batch effect, as experiment 1 was run separately from experiments 2 and 3. This batch effect was apparent in a density plot of the average probe intensities, which displayed a shift between the two runs (Additional file 2B). However, differences between the gene expression profiles of the PAXgene and Tempus sampling systems were the second most important source of variation in the data (PC2, Additional file 2A). We explored the PCA up to six components (together explaining 71% of the variation in the data) without finding a pattern distinguishing the original protocols from the modified protocol, or distinguishing between samples with low or high quality and quantity RNA (results not shown).

#### Comparison of RNA gene expression profiles from PAXgene and Tempus tubes in combination with their original protocols and the modified protocol

To assess differences in gene expression between PAXgene and Tempus tubes when used with the original and modified protocols, we analysed five contrasts from the three experiments (Fig. 1; Table 2). In experiment 1, blood from the same individual was sampled on both PAXgene and Tempus tubes, and yet signals from up to 3143 microarray probes differed significantly between the sampling systems (p < 0.05, Table 1), supporting earlier findings [14–16]. In our analyses of all 5 contrasts, we found that signals from between 1440 probes (1346 genes) to 7142 probes (6250 genes) differed significantly between PAXgene and Tempus systems (p < 0.05, Table 1).

**Table 2:** Comparisons of results from statistical analyses (contrasts), “Focus of analysis” explains the focus of the comparison

Focus of analysis	Contrasts compared	Direction		Pearson	Spearman
		Same	Opposite		
Protocols used in exp. 1	c1 vs c2	1066	0	0.786	0.444
Protocols used in exp. 2	c3 vs c4	887	0	0.854	0.617
Reproducibility of the original protocol between exp. 1 and exp. 2	c1 vs c3	708	3	0.563	0.288
Reproducibility of the modified protocol between exp. 1 and exp. 2	c2 vs c4	417	0	0.604	0.222
Reproducibility of the modified protocol between exp. 1 and exp. 3	c2 vs c5	1199	8	0.681	0.313
Reproducibility of the modified protocol between exp. 2 and exp. 3	c4 vs c5	1330	0	0.857	0.487

“Comparison” specifies contrasts used in the comparison; see Table 1 and Fig. 1. “Direction” specifies if the significant probes (p < 0.05, Table 1) are expressed in the same or in the opposite direction between contrasts; hence, “Same” gives the number of probes where the logFC was positive or negative in both contrasts; “Opposite” gives the number of probes where the logFC was positive in one contrast and negative in the other. “Pearson” and “Spearman” give the Pearson and Spearman correlations, respectively, for the logFC values of all probes in the two contrasts used in the comparison

**Fig. 3:**
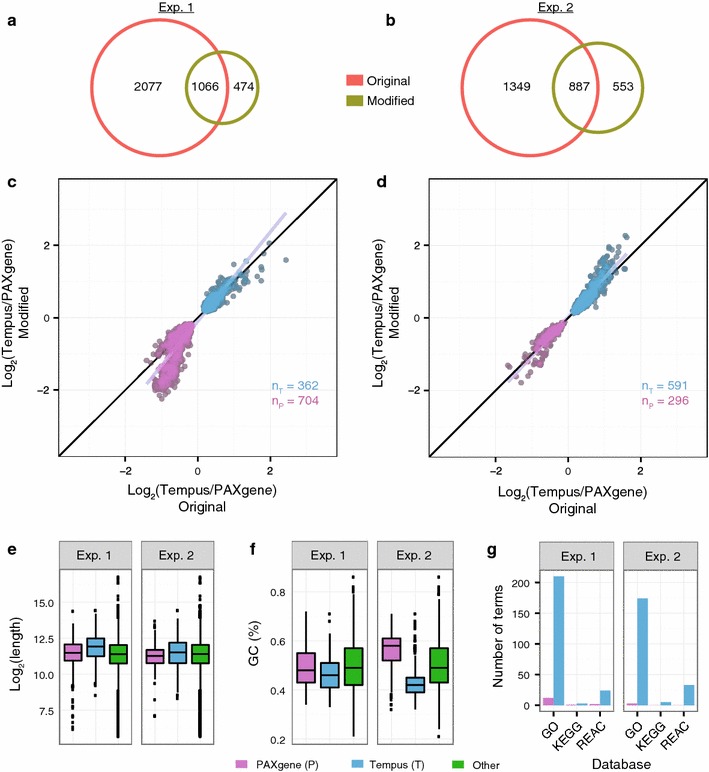
Comparison of protocols. **a**, **b** Venn diagrams showing the number of microarray probes having significantly different signals (p < 0.05) between PAXgene and Tempus systems for the original protocols (*orange*) and the modified protocol (*olive*) in experiment 1 (**a**) and experiment 2 (**b**). **c**, **d** Scatter plots showing the logFC values of the 1066 and 887 significant probes found in common between the protocols in experiment 1 (**c**) and in experiment 2 (**d**), respectively. *Grey* and *black lines* are linear regression fits to the data and idealized regression lines (logFC original = logFC modified), respectively. **e** Length and **f** GC content of transcripts preserved in PAXgene (logFC <0; *purple*) and in Tempus (logFC >0; *blue*) from **c** and **d**. All other transcripts (“Other”; *green*) are included as reference. See Fig. 2 for explanation of graphs. **g** The number of significantly enriched biological terms for transcripts preserved in the PAXgene and Tempus tubes

The number of probes that were differently expressed between the sampling systems was significantly reduced when the modified protocol was applied instead of the original protocols in experiment 1 and 2 (p = 4.6e−07 and p = 0.02, respectively). When comparing the significantly differentially expressed probes (p < 0.05) for the original and modified protocols, we found 1066 probes in common in experiment 1 (Fig. 3a), and 887 probes in common in experiment 2 (Fig. 3b). For both experiments, these common probes were similarly affected by the sampling systems. Specifically, all the significant probes common for the original and modified protocols, in both experiments, had either consistent positive, or consistent negative, logFC values (Fig. 3c, d). As we used the Tempus signal as reference, a positive logFC (log_2_ Tempus/PAX >0) implied that the level of the probe’s target transcript was higher in Tempus tubes (preserved in Tempus), whereas a negative logFC (log_2_ Tempus/PAX <0) implied that the level of the probe’s target transcript was higher in PAXgene tubes (preserved in PAXgene).

The consistent changes in logFC values for the common probes suggested that the target transcripts for these probes were affected by differences in the sampling systems, rather than by differences in the isolation protocols (Fig. 3c, d). We speculated that physical properties of these transcripts might explain some of the differences, and investigated the GC content and length of their respective FASTA sequences. Transcripts preserved in PAXgene were shorter and had a higher GC content compared to those preserved in Tempus and all other annotated transcripts (Fig. 3e, f, respectively). In addition, the transcripts preserved in Tempus were significantly overrepresented among several terms in the GO, KEGG and REACTOME databases, such as “activation and aggregation of platelets” and 52 metabolic processes, whereas only a few such terms (“Generic Transcription Pathway”, “gene expression” and 6 metabolic processes) were significant for the transcripts preserved in PAXgene (Fig. 3g). Consequently, the transcripts preserved in Tempus appear to have distinct physical and functional characteristics, whereas transcripts preserved in PAXgene appear to be more similar to the transcriptomic background.

Among the probe targets consistently preserved in PAXgene (logFC <0) across protocols, a set of probes (n = 380) had lower logFC values for the modified protocol compared to the original protocols (Fig. 3c; points below the regression line). Although the transcripts targeted by these probes may be more affected by the modified protocol, these transcripts were not significantly different from the majority of transcripts preserved in PAXgene in regard to GC content and transcript length. Furthermore, these transcripts were associated with only one significant REACTOME term (“Generic Transcription Pathway”).

In summary, using the modified protocol reduces the number of probes significantly differentially expressed between the PAXgene and Tempus systems. Nevertheless, there are several transcripts that differ consistently between the two systems, independent of RNA isolation protocol. Both physical and biological properties of the transcripts appear to be relevant for the gene expression differences observed between PAXgene and Tempus.

#### Reproducible gene expression profiles from PAXgene and Tempus tubes in combination with their original protocols and the modified protocol

To assess to what extent the differences in gene expression between sampling systems were reproducible between different biological samples, we analysed the five comparisons from all three experiments (Fig. 1; Table 1). We defined reproducible probes to have significantly different signals (p < 0.05) between PAXgene and Tempus systems in at least two experiments.

**Fig. 4:**
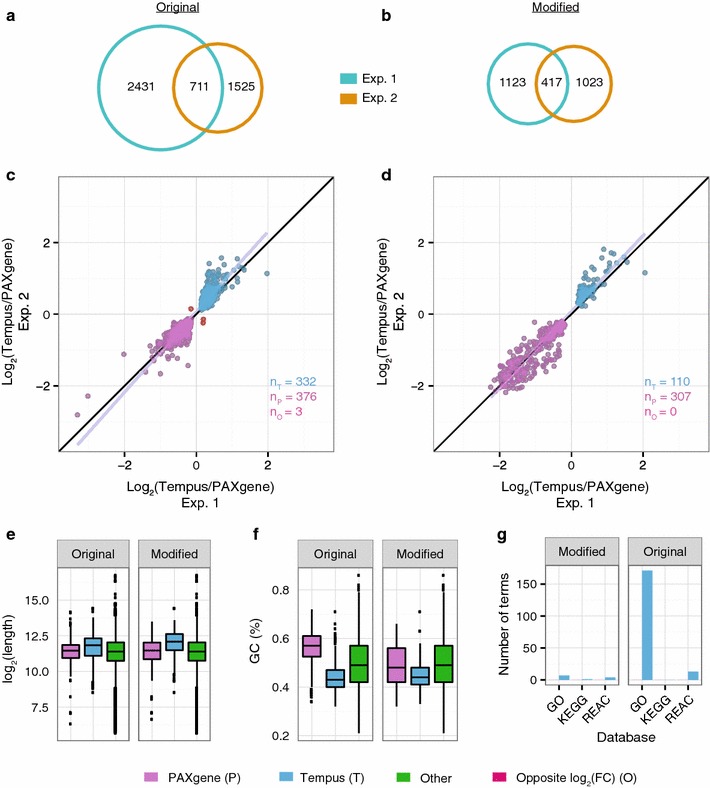
Reproducibility. **a**, **b** Venn diagrams showing the overlap in microarray probes having significantly different signals (p < 0.05) between PAXgene and Tempus systems for experiment 1 (*turquoise*) and experiment 2 (*orange*) for the original protocols (**a**) and the modified protocol (**b**). **c**, **d** Scatter plots showing the logFC values of the 711 and 417 significant probes found in common between experiment 1 and 2 for the original protocols (**c**) and for the modified protocol (**d**), respectively. **e** Length, **f** GC content, and **g** number of significantly enriched biological terms of transcripts preserved in PAXgene (*purple*) and in Tempus (*blue*) from **c** and **d**; see Fig. 2 for additional details

When comparing experiment 1 and 2, the number of reproducible probes for the modified protocol (417; Table 2; Fig. 4a) was comparable to the number of reproducible probes for the original protocols (711, Table 2; Fig. 4b). Again, we found these common probes to be similarly affected by the sampling systems regardless of protocol (Fig. 4c, d). Similar to the transcripts showing consistent changes irrespective of protocol, the transcripts that were reproducibly preserved in the PAXgene tubes (logFC <0) were shorter and had a higher GC content than those preserved in the Tempus tubes (logFC >0; Fig. 4e, f, respectively). We also found the results from the GO, KEGG, and REACTOME databases to be in line with our earlier findings. Transcripts preserved in Tempus were overrepresented in number of significant terms compared with transcripts conserved in PAXgene (Fig. 4g).

**Fig. 5:**
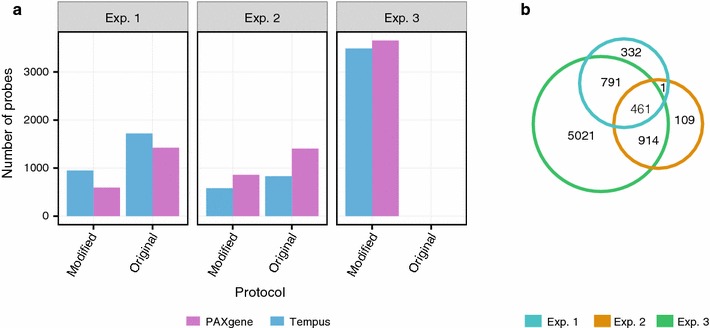
Comparison of the significant probes across the three experiments. **a** Number of probes preserved in PAXgene (logFC <0; *purple*) and Tempus (logFC >0; *blue*) for the original protocols and the modified protocol in all three experiments. **b** Venn diagram of microarray probes having significantly different signals (p < 0.05) between PAXgene and Tempus for the modified protocol across all three experiments

**Fig. 6:**
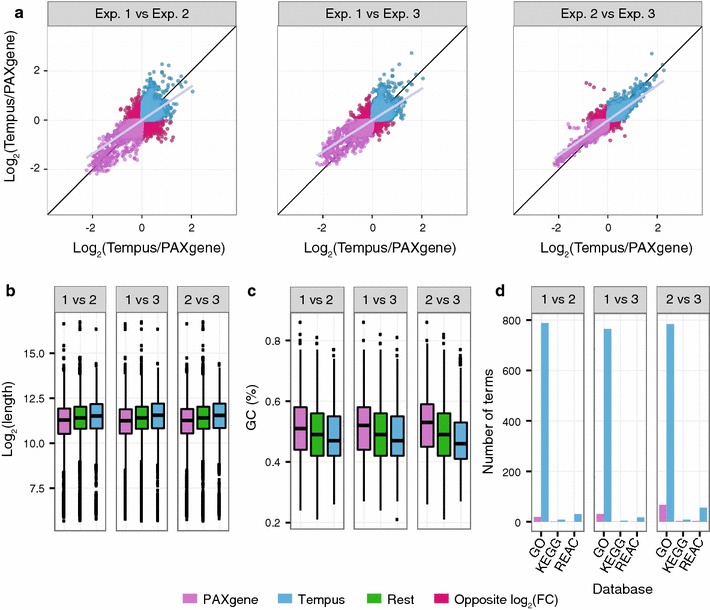
Signal changes and physical characteristics of all probes present on the Illumina HT-12 v4 chip. **a** Scatter plots showing for all probes on the Illumina HT-12 v4 chip, their logFC values from the comparisons of PAXgene and Tempus with the modified protocol on all three experiments. **b** Length, **c** GC content, and **d** number of significantly enriched biological terms of transcripts consistently preserved in PAXgene (*purple*) and Tempus (*blue*) from (**a**)

As experiment 3 included a larger set of samples than experiments 1 and 2 (61, 4 and 13, respectively), we expected to have a higher statistical power to identify differences between PAXgene and Tempus. Indeed, we found more than four times as many significantly differentially expressed probes in experiment 3 as in experiments 1 or 2 (Fig. 5a; Table 1). This larger set of probes included most of the significant probes from experiment 1 and 2 (Fig. 5b), suggesting that most of the differences in significant probes between these two experiments were due to lack of statistical power. To further investigate this possibility, we compared how all the 47323 probes on the Illumina chip (HT-12 v4) were affected by the sampling systems. The logFC values for all probes were highly correlated between experiments (Fig. 6a; Pearson’s r = 0.60–0.86, Additional file 3; Pearson’s r = 0.56) and most of the probes with high absolute logFC values were similarly affected between experiments. We also found the physical characteristics of the transcripts preserved in PAXgene (logFC <0) to differ from the transcripts preserved in Tempus (logFC >0), in agreement with our earlier findings (Fig. 6b–d).

These results indicate that the differences between PAXgene and Tempus systems affect many probes, and that there is a high degree of correlation between experiments. To further test to what extent these differences were reproducible across studies, we compared the results obtained with the original protocols (experiment 1) with data from a previous study by Menke et al. [14]. Despite some major differences with regard to study setup, such as subject inclusion (only men were studied by Menke et al.), geography, time of study, and version of chip used for gene expression analysis, the effects of the sampling systems were similar for the two experiments (Additional file 4; Pearson’s r = 0.27).

Collectively, these results indicate that the technical differences between PAXgene and Tempus systems are highly reproducible between different biological samples and experiments. In turn, this reproducibility suggests that the technical influence of the sampling systems on the gene expression profiles can be accounted for by running a control study in parallel with biologically relevant samples. The control study should contain RNA obtained from individuals sampled on both PAXgene and Tempus tubes, which should to be isolated and analysed together with the biologically relevant samples. By doing so, the technical differences may be estimated and subsequently accounted for to obtain biologically relevant gene expression profiles.

## Conclusions

Several studies have claimed the two most common RNA stabilizing blood-sampling systems—PAXgene and Tempus—to have incomparable RNA expression profiles. Similar to previous studies, we found that blood from the same individuals sampled on the PAXgene and Tempus systems resulted in more than 2000 genes with significantly different expression profiles between the systems. Nevertheless, by developing and using a protocol applicable to both PAXgene and Tempus tubes, we significantly reduced the number of genes that differed between the two systems (p = 4.6e−07). Moreover, we found the modified protocol to improve the miRNA yield from the PAXgene system, which had the lowest miRNA output with the original protocol. Regardless of protocol used, however, our results indicate that the expression profile for a large fraction of genes is affected by the sampling systems. These expression profile differences were highly correlated between protocols and were also highly correlated with the differences we found when we measured blood gene expression profiles of different individuals from two independent biobanks based on the PAXgene and Tempus systems. Consequently, whereas the differences between the sampling systems affect a large set of genes, our results show that these differences are exceedingly reproducible—at least when the different samples are analysed within the same lab. We therefore propose that technical differences between PAXgene and Tempus, when both systems are used in the same study, can be handled by running a control experiment with identical samples on both sampling systems. The gene expression differences identified in such a control experiment can then be used to correct for technical differences when analysing biologically relevant samples from the two sample systems. We expect that this approach will make additional cohorts available for large-scale replication studies or clinical gene expression research to identify robust, disease related biomarkers.

## Methods

### Study design

We did three experiments (Fig. 1) to investigate whether the modified protocol would reduce the differences in gene expression between the PAXgene and Tempus sampling systems. Venous blood was drawn from healthy volunteers on both sampling systems and RNA was isolated by the use of the original protocols and/or the modified protocol. Since this was a study of technical issues and of no medical interest, the Regional Ethics Committee (REK) concluded that no approval from the committee was required for this part of the study (REK: 2013/2422-2).

#### Experiment 1

Venous blood was collected by phlebotomy with butterfly needle from four healthy female volunteers (aged 26–34) with their consent. Sampling was performed after 2 h of fasting and abstention from coffee, medication and exercise. Blood was collected into PAXgene tubes **(**2.5 mL blood + 6.9 mL buffer) and Tempus tubes (3 mL blood + 6 mL buffer). The first drawn tube from all participants was discarded as recommended by the PAXgene protocol. All samples were subsequently inverted 10 times before being stored at −80 °C. Before isolation of RNA, the tubes were thawed at room temperature for 16 h as recommended by the PAXgene protocol for enhanced yields. The content of each tube was split in two aliquots, where one aliquot was isolated using the sampling systems’ original protocol and the second aliquot with the modified protocol (described below).

#### Experiment 2

Samples from healthy volunteers (aged 50–82) from two different biobanks were used. The Norwegian Women and Cancer Cohort (NOWAC) provided blood from six healthy females drawn on PAXgene tubes and the Health Survey of North-Trøndelag (HUNT3) provided blood from seven healthy females drawn on Tempus tubes. All samples arrived at our facility frozen and were stored at −80 °C. Before RNA isolation the tubes were thawed for 16 h at room temperature. Each tube was split in three aliquots and RNA was isolated with the sampling systems’ original protocols as well as the modified protocol.

#### Experiment 3

Samples from the same biobanks as in experiment 2 were used, but with a larger sample set: n = 41 (NOWAC) and n = 20 (HUNT3), all females aged between 43 and 70. RNA was isolated from the samples using the modified protocol, and otherwise treated the same way as in experiment 2.

### Sample treatment and RNA isolation

Both the original protocols and the modified protocol are divided into four parts: (1) buffer removal, (2) pellet washing, (3) running through column and (4) elution. Samples isolated with the modified protocol and the original PAXgene protocol were treated with DNase to remove any traces of genomic DNA.

#### Original RNA extraction protocols

RNA from samples collected on PAXgene tubes was isolated according to the “Purification of Total RNA from human whole Blood Collected into PAXgene Blood RNA Tubes” protocol in the PAXgene Blood RNA Kit Handbook (PreAnalytiX GmbH, 08/2005, REF: 762174). The Tempus tubes were processed according to the “Tempus Spin RNA Isolation Kit” protocol (Applied Biosystems, 2008, Part Number 4379232 Rev. D).

#### Modified RNA extraction protocol

In the modified protocol (Additional file 5), total content in the tubes (blood and preservative in either PAXgene or Tempus) was diluted with 1× PBS (1:1 vol of blood: 1× PBS). The tubes were subsequently vortexed for 30 s before centrifugation for 60 min (4500 rcf, 4 °C) using a swing out rotor. The supernatant was removed and the tubes were decanted upside down for 2 min to dry. Buffer 1 (350 μL) from the PAXgene kit was added and the pellet was dissolved by pipetting up and down. The samples were transferred to sterile 1.5 mL tubes (Eppendorf AG, Hamburg, Germany) before adding Buffer 2 (300 μL) and proteinase K (40 μL), both from the PAXgene kit. The samples were vortexed and incubated for 10 min at 55 °C using a shaker incubator (1200 rpm, Eppendorf thermomixer comfort, Eppendorf). After a quick spin down, the lysate was directly pipetted to a PAXgene spin column and centrifuged for 3 min (1500 rcf). The supernatant was transferred to a new tube and 100% ethanol (812 μL, 1.25 times the present volume) was added and mixed by turning the tube upside down. The lysate- and ethanol mix was transferred to filter from the mirVana kit (Life Technologies, Part Number AM1560), centrifuged for 15 s (9000 rcf) and the flow-through was discarded. Since the filter has a maximum volume of 700 μL, this process was repeated until all the mix was run through. Wash solution 1 (700 μL) from the mirVana kit (Life Technologies) was added and the samples were centrifuged for 15 s (9000 rcf). The flow-through was subsequently discarded before 500 μL Wash solution 2/3 (Life Technologies) was added. The samples were again centrifuged for 15 s (9000 rcf). The flow-through was discarded before treating with DNase1 (80 µL) (PreAnalytiX) for 15 min at room temperature. The filter was washed with a second round of Wash solution 2/3 (500 μL), and centrifuged for 15 s (9000 rcf). The filter was dried for one min by centrifuging (9000 rcf) before it was transferred to a new collection tube. To elute the RNA 50, μL pre-heated nuclease free water (Tempus kit, Applied Biosystems) was added followed by spinning for 30 s (9000 rcf). The elution step was repeated and the tubes were centrifuged for 2 min. The eluate was transferred to a new sterile 1.5 mL tube (Eppendorf AG) without disturbing the debris. Finally, the samples were incubated at 65 °C for 5 min before RNA yield and quality were measured. All samples were stored at −80 °C.

### RNA quality check

The concentration (OD260) and purity (OD260/280 ratio) of extracted total RNA was measured using NanoDrop ND-1000 spectrophotometer (Thermo Fisher Scientific, MA, USA). Agilent 2100 Bioanalyzer **(**Agilent Technologies, Santa Clara, CA, USA) was used to assess the RNA integrity using the Eukaryote total RNA 6000 Pico LabChip kit and the Eukaryote total RNA Pico assay according to the manufacturer’s instructions. The RNA integrity numbers (RIN) were calculated using the Agilent 2100 Expert Software (Agilent Technologies); RIN = 1 indicates low RNA quality and RIN = 10 indicates highest RNA quality.

### miRNA detection

Samples from experiment 1 were used to verify the presence of miRNA by the use of TaqMan-qPCR (Applied Biosystems). miRNA was detected by running quantitative real-time PCR (qRT-PCR) on all samples isolated from the volunteers. A serial dilution of cervical adenocarcinoma (HeLa-S3) total RNA (Ambion Life Technologies, cat. Nr: AM7852) was used to make a standard curve (range: 200 to 0.02 ng/µL). Total RNA (40 ng) was reverse transcribed in a 15 µL reaction using TaqMan reverse transcription reagents (Applied Biosystems). The TaqMan MicroRNA Assay IDs 000391, 001098, and 002626 (Applied Biosystems) were used to quantify the expression of hsa-miR-16, hsa-miR-181, and hsa-miR-423, respectively. Quantitative PCR was carried out on a StepOnePlus Real-Time PCR System (Applied Biosystem). The concentration (ng/µL) of miR-16, miR-181 and miR-423 in the blood samples was calculated from the standard curve equation.

### Microarray processing

The Illumina TotalPrep RNA Amplification Kit (Ambion Inc., Austin, TX, USA) was used to amplify RNA for hybridization on Illumina BeadChips. The three experiments used in this study were processed in two separate runs. Experiment 1 was run separately from experiment 2 and 3. Total RNA was used in the first strand cDNA synthesis by reverse transcription. Following the second strand cDNA synthesis and cDNA purification steps, in vitro transcription to synthesize cRNA was carried out for 12 h. Biotin-labeled cRNA was hybridised to Illumina HumanHT-12 v4 Expression BeadChips (Illumina, Inc., San Diego, CA, USA) according to the manufacturer´s protocol. Microarrays were scanned with the BeadArray Reader (Illumina).

### Data and statistical analysis

A total of 106 samples including 3 technical replicates were analysed. Illumina BeadArray studio (Illumina) was used to handle Illumina data. Data analysis was performed using R (http://cran.r-project.org), together with tools from the Bioconductor project (http://bioconductor.org), Galaxy (http//usegalaxy.org) and UCSC (https://genome.ucsc.edu/cgi-bin/hgTables). Data from 47323 probes was transformed and normalized using quantile–quantile normalization. For differential expression analysis, functions from the limma package [17] were used. The Benjamini-Hochberg procedure was used to correct for multiple testing. We used a paired analysis on the data from the samples in experiment 1 and non-paired analyses of the data from experiments 2 and 3. Data from Menke et al. [14], was downloaded from (https://www.ebi.ac.uk/arrayexpress/help/GEO_data.html). We used a subset of their data from samples isolated with the original protocols and without dexamethasone treatment. These data was used in a non-paired analysis against our samples from experience 1 isolated with the original protocols.

Packages ggplot2 [18], reshape2 [19], gridExtra [20] and Vennerable [21] were used for data visualization. Enrichment analysis of lists of the probes of interest were analysed by gProfiler [22].

Statistical significance of differences between probes reproducible across experiments and between different sample sets was calculated using Fisher´s exact test. Significance of differences (p < 0.05) in correlation between average expression as well as logFC between experiment 1 and 2 for both protocols were calculated with Pearson and Spearman equations. Significance of differences between the relative expression of miR-16, miR-181, and miR-423 between the tubes and protocols was calculated using a two-sided paired Student’s *t* test.

When describing quantity changes from an initial to a final value we use log_2_ fold change (logFC) throughout this article.

The five contrasts produced from the statistical analyses were compared with regard to protocols (original protocols versus the modified protocol), and with regard to reproducibility (each protocol across two and three experiments) (Fig. 1; Table 2; Additional file 6).

### Transcript length, GC content and biological terms

To investigate length and GC content of transcripts of interest, gene symbols for all Illumina ProbeIDs were retrieved from the microarray’s Bioconductor annotation package (illuminaHumanv4.db). Transcript IDs from the RefSeq database were then retrieved from the UCSC Table Browser (https://genome.ucsc.edu/cgi-bin/hgTables) by uploading and intersecting the gene symbols with the refGene table. In total, the UCSC Table Browser identified 39818 of the 47323 probes. The resulting list of RefSeq IDs was exported to Galaxy (http://usegalaxy.org) for further analyses. Galaxy produced the FASTA sequence of the respective RNA sequence of the imported genes (transcripts). The “geecee” tool was used to calculate the GC content of each FASTA sequence, and the “FASTA manipulation” tool was used to calculate the length of each FASTA sequence. The gProfiler package in R was used to identify whether differentially expressed genes were significantly enriched within biological terms in the GO, KEGG, and REACTOME databases.

## Additional files

**Additional file 1.** Overview of all samples used in this study and their respective information.

**Additional file 2.** Principal component analysis (PCA) and probe signal distributions. (A) Samples plotted in the plane defined by the first (PC1) and second (PC2) principal components from a PCA analysis of all the gene expression data. Differences between the first and the second microarray run are shown as the first component in the PCA, explaining 39% of the differences in the samples due to batch effects. The second component reveals that differences between the sampling systems contribute 14% of the differences between the samples. (B) Density plot of the probe signals from the first and second microarray run. There is a clear shift in the probe signal distribution, seen as a shift in the peaks, between the two runs.

**Additional file 3.** Behaviour of all probes present on the Illumina HT-12 v4 chip. LogFC values from the analysis of PAXgene and Tempus in combination with the original protocol of all the probes present on the Illumina HT-12 v4 chip are compared between experiment 1 and 2.

**Additional file 4.** Comparison of logFC values between experiment 1 and the study by Menke et al. [4]. Scatter plot of the logFC values from experiment 1 when the original protocols were used and the logFC values when the original protocols were used in the Menke et al. study. The plot includes all probes that were common between this study and Menke et al.

**Additional file 5.** Flowchart of the modified protocol. The modified protocol consists of three parts: (**A**) collecting blood, (**B**) processing stabilized blood, and (**C**) isolating RNA. The overview of the protocol is outlined in the first column (“Process”), and the reagents for each step is given in the second column (“Reagents”). The modified protocol is assembled from three kits: Tempus (blue), PAXgene (cerise), and mirVana (green); note that the reagents used from the Tempus kit are universal and can be replaced with equivalent reagents from other suppliers.

**Additional file 6.** Tables of probes found significant between PAXgene and Tempus. The workbook contains 5 sheets of tables, one for each contrast (Fig. 1). Each table is the output from the function topTable from limma and contains all significant probes identified in the contrast. The columns are the probe ID (ProbeID); the gene symbol for the gene targeted by the probe (TargetID); the log_2_ fold change (logFC) of the Tempus–PAXgene contrast; the average probe signal (AveExpr); the moderated t-statistic (t), corresponding p value (P.Value), and Benjamin-Hochberg adjusted p-value (adj. P. Val); the log-odds that the gene is differentially expressed (B); and the Illumina-specific probe ID (ilmnid).
