# Supplementary figures and images for: Gene expression differences between PAXgene and Tempus blood RNA tubes are highly reproducible between independent samples and biobanks
Source: BMC Res Notes. 2017 Mar 23;10:136. doi: 10.1186/s13104-017-2455-6 (PMC5364635; doi:10.1186/s13104-017-2455-6)

**A**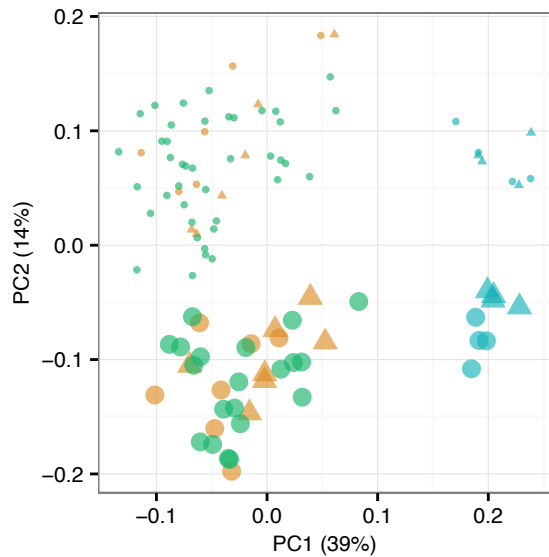

Experiment

- 1
- 2
- 3

Protocol

- Modified
- Original

Tube

- PAXgene
- Tempus

**B**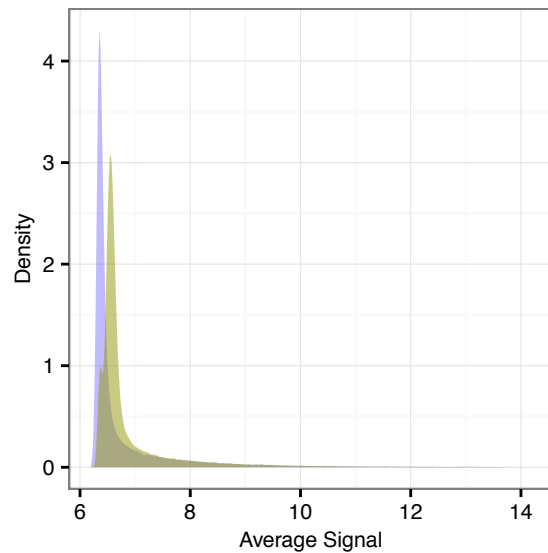

Run

- First
- Second

Supplement: Supplementary file 2 — Additional file 2. Principal component analysis (PCA) and probe signal distributions. (A) Samples plotted in the plane defined by the first (PC1) and second (PC2) principal components from a PCA analysis of all the gene expression data. Differences between the first and the second microarray run are shown as the first component in the PCA, explaining 39% of the differences in the samples due to batch effects. The second component reveals that differences between the sampling systems contribute 14% of the differences between the samples. (B) Density plot of the probe signals from the first and second microarray run. There is a clear shift in the probe signal distribution, seen as a shift in the peaks, between the two runs. [file 13104_2017_2455_MOESM2_ESM.pdf]

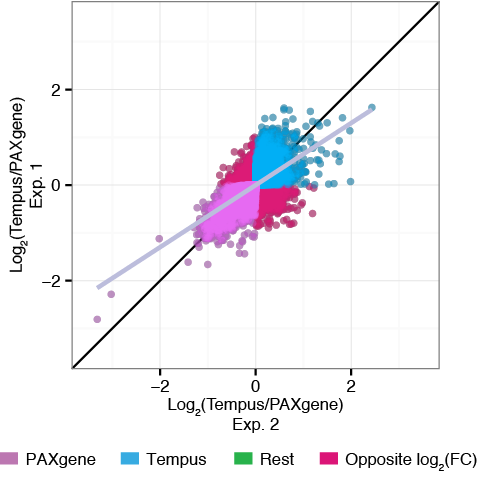

Supplement: Supplementary file 3 — Additional file 3. Behaviour of all probes present on the Illumina HT-12 v4 chip. LogFC values from the analysis of PAXgene and Tempus in combination with the original protocol of all the probes present on the Illumina HT-12 v4 chip are compared between experiment 1 and 2. [file 13104_2017_2455_MOESM3_ESM.png]

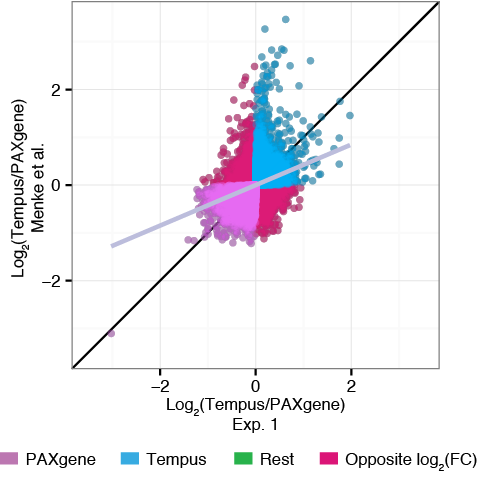

Supplement: Supplementary file 4 — Additional file 4. Comparison of logFC values between experiment 1 and the study by Menke et al. [4]. Scatter plot of the logFC values from experiment 1 when the original protocols were used and the logFC values when the original protocols were used in the Menke et al. study. The plot includes all probes that were common between this study and Menke et al. [file 13104_2017_2455_MOESM4_ESM.png]

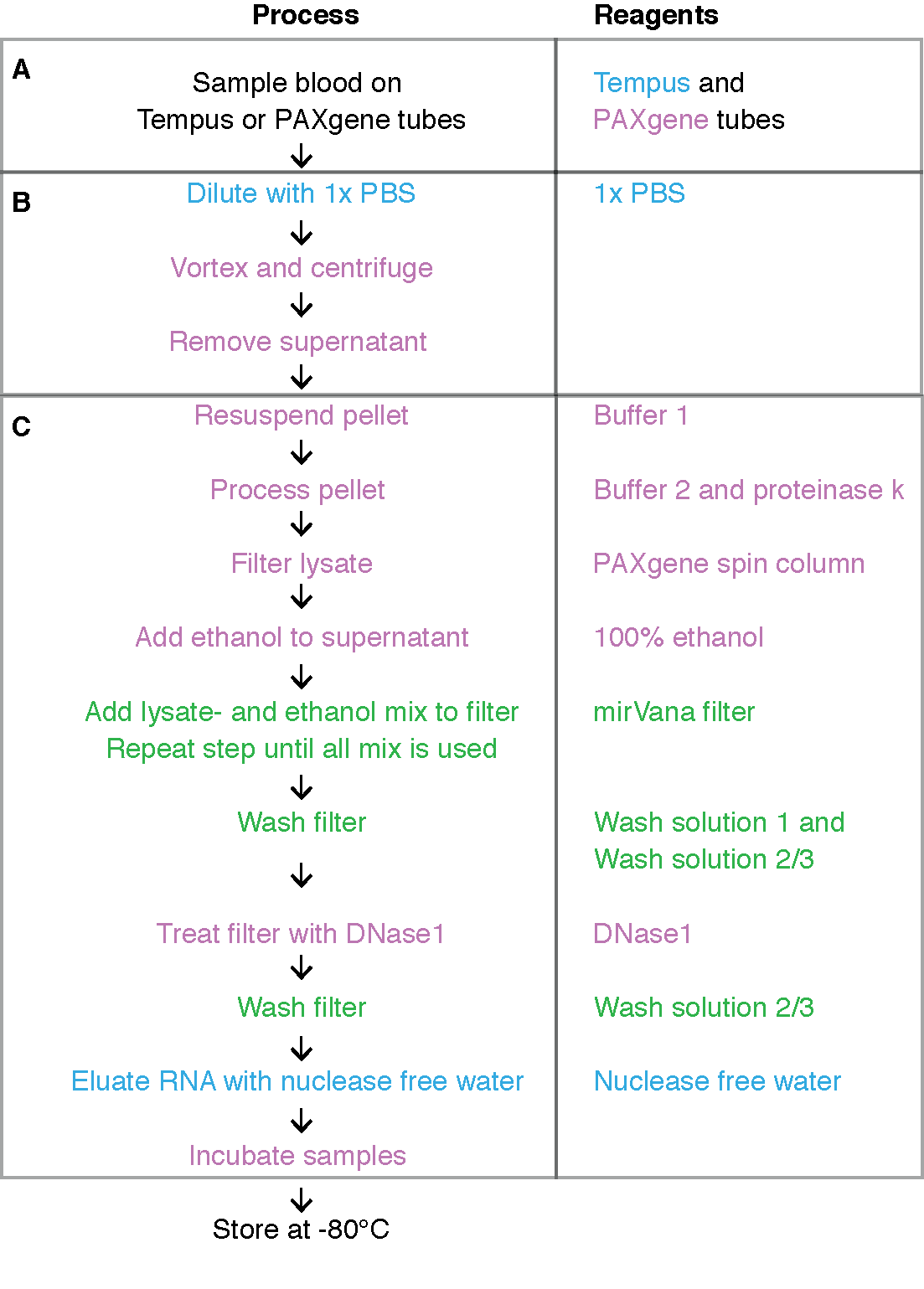

Supplement: Supplementary file 6 — Additional file 6. Tables of probes found significant between PAXgene and Tempus. The workbook contains 5 sheets of tables, one for each contrast (Fig. 1). Each table is the output from the function topTable from limma and contains all significant probes identified in the contrast. The columns are the probe ID (ProbeID); the gene symbol for the gene targeted by the probe (TargetID); the log2 fold change (logFC) of the Tempus–PAXgene contrast; the average probe signal (AveExpr); the moderated t-statistic (t), corresponding p value (P.Value), and Benjamin-Hochberg adjusted p-value (adj. P. Val); the log-odds that the gene is differentially expressed (B); and the Illumina-specific probe ID (ilmnid). [file 13104_2017_2455_MOESM6_ESM.png]
